# OP67 “Black Box Bottleneck” Paradigm And Transparency Issues On Artificial-Intelligence-Based Tools In Health Technology Assessment: A Scoping Review

**DOI:** 10.1017/S0266462324001260

**Published:** 2025-01-07

**Authors:** Denis Satoshi Komoda, Marilia Mastrocolla de Almeida Cardoso, Ana Renata Lima, Marília Berlofa Visacri, Carlos Roberto Silveira Correa, Brígida Dias Fernandes

## Abstract

**Introduction:**

One of the pillars of health technology assessment (HTA) is transparency, which guarantees reproducibility and accountability. Due to the “black-boxness” of artificial intelligence (AI) models, the use of AI-based tools adds new layers of complexity for transparency issues. The aim of this scoping review is to map AI-based tools applied in HTA processes, regarding human supervision and “open-sourceness” aspects.

**Methods:**

A search strategy using the terms “AI,” “HTA,” and correlated terms was performed in nine specialized databases (health and informatics) in February 2022. Inclusion criteria were publications testing AI models applied in HTA. Selection of studies was performed by two independent researchers. No filter was applied. Variables of interest included a subset of AI models (e.g., machine learning [ML], neural network), learning methods (e.g., supervised, unsupervised, or semi-supervised learning), and code availability (e.g., open source, closed source). Data were analyzed exploratorily as frequency statistics.

**Results:**

ML with one layer of hidden nodes was applied in 48 (78.6 %) studies, while deep learning (DL) (two-plus layers) were applied in eight (13.1 %). ML models that used supervised learning accounted only for half of the reported models, while half used unsupervised learning. Considering supervision methods in DL models, seven used unsupervised learning, and one used supervision. Four studies did not report the AI model, and 14 studies did not report the supervision paradigm. It was not possible to assess “open-sourceness” in 31 studies. Among the identified software, seven models were not open source, and 13 were open source.

**Conclusions:**

Transparency and accountability are of utmost importance to HTA. Complexity of AI models may introduce trustworthiness issues in HTA. Transparency provided by open-source code becomes essential in building trust in the automation of HTA processes, as does quality of report. Although progress has been observed in transparency and quality, the lack of a methodological framework still poses challenges in the field.

